# SN38 loaded nanostructured lipid carriers (NLCs); preparation and in vitro evaluations against glioblastoma

**DOI:** 10.1007/s10856-021-06538-2

**Published:** 2021-06-30

**Authors:** Ali Sabouri Shirazi, Reyhaneh Varshochian, Mahsa Rezaei, Yalda Hosseinzadeh Ardakani, Rassoul Dinarvand

**Affiliations:** 1 Department of Pharmaceutics, Faculty of Pharmacy, Tehran University of Medical Sciences, Tehran, Iran; 2 Nanotechnology Research Centre, Faculty of Pharmacy, Tehran University of Medical Sciences, Tehran, Iran; 3 Department of Pharmaceutics, School of pharmacy, Shahid Beheshti University of Medical Sciences, Tehran, Iran; 4 School of chemistry, College of Science, University of Tehran, Tehran, Iran; 5 Department of Pharmaceutics, Biopharmaceutics and Pharmacokinetics Division, Faculty of Pharmacy, Tehran University of Medical Sciences, Tehran, Iran

## Abstract

SN38 is the active metabolite of irinotecan with 1000-fold greater cytotoxicity compared to the parent drug. Despite the potential, its application as a drug is still seriously limited due to its stability concerns and low solubility in acceptable pharmaceutical solvents. To address these drawbacks here nanostructured lipid carrier (NLC) containing SN38 was prepared and its cytotoxicity against U87MG glioblastoma cell line was investigated. The formulations were prepared using hot ultrasonication and solvent evaporation/emulsification methods. NLCs with a mean size of 140 nm and particle size distribution (PDI) of 0.25 were obtained. The average loading efficiency was 9.5% and its entrapment efficiency was 81%. In order to obtain an accurate determination of released amount of SN38 a novel medium and extraction method was designed, which lead to an appropriate in vitro release profile of the drug from the prepared NLCs. The MTT test results revealed the significant higher cytotoxicity of NLCs on U87MG human glioblastoma cell line compared with the free drug. The confocal microscopy images confirmed the proper penetration of the nanostructures into the cells within the first 4 h. Consequently, the results indicated promising potentials of the prepared NLCs as a novel treatment for glioblastoma.

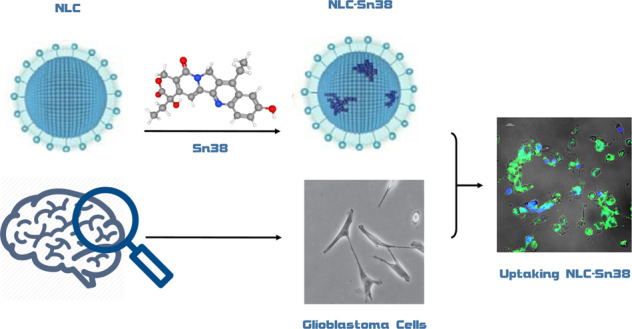

## Introduction

Glioblastoma multiform (GBM), accounting about two third of all malignant glioma cases, is the deadliest brain tumor with a 5-year survival rate of <5%. Surgical resection, the most effective and reliable treatment of GBM, is mostly limited to certain cases in which the tumor’s location is accessible [1, 2]. Furthermore, penetration of chemotherapeutic drugs to their site of action in central nervous system (CNS) is mostly restricted by the blood-brain barrier (BBB). This may explain partly why the application of chemotherapeutics even in combination with surgical resections is still ineffective in some cases. An effective approach to tackling this problem is the application of novel drug delivery systems including polymer-based nanoparticles (NPs), liposomes, dendrimers, and lipid-based NPs [3].

The capability of passing BBB as well as the highly biocompatible and biodegradable nature of lipid-based NPs has made them as promising and efficient carriers in CNS drug delivery [4]. Owing to the unique physiochemical properties of lipids, lipid-based NPs can be easily prepared by recrystallization or coacervation of previously emulsified molten or dissolved lipids [5, 6]. Two frequently applied forms of lipid-based NPs include solid lipid NPs (SLNs) and nanostructured lipid carriers (NLCs). Lipids that are applied in the formulation of SLNs are in the solid state; however, a mixture of liquid and solid lipids are utilized in the formation of NLCs. The main advantages of NLCs over SLNs are the higher drug loading capacity and diminished drug leakage during storage period [7, 8].

The cytotoxic compound, 7- ethyl- 10 hydroxy camptothecin which is also known as SN38 is the active metabolite of irinotecan, a chemotherapeutic agent belonging to camptothecin family [9]. In 1996, FDA approved application of SN38 for treatment of metastatic colorectal carcinoma for the first time [10]. In spite of 100–1000 times more potency compared to parent compound, only 1–9% of injected irinotecan dose would be converted to SN38 through the activity of carboxylesterase enzyme located in the liver [11]. Accordingly, nowadays, administration of SN38 instead of irinotecan has attracted attention of many researchers for treatment of different malignancies such as breast, lung, colon, and ovarian cancers [12–16].

Despite the great potency of SN38 in treatment of different malignancies, its poor solubility in aqueous solvents with or without pharmaceutically acceptable surfactant/co-surfactant/co-solvent (e.g., polysorbate 80, cremophor, etc.) has mostly restricted its clinical application. Furthermore, camptothecins are reported to be unusable at high pH values. It has been shown that at pHs higher than 5 (for instance the physiological pH) the lactone ring which is essential integrity for being pharmacologically active, will open and results in an inactive carboxylated form of the drug [17–19]. Recently, many nanoscale drug delivery systems including polymeric compounds, liposomes, micelles, and dendrimers have been successfully applied for effective delivery of SN38 and overcoming the problems associated with its delivery such as low circulation half-life, solubility, and the cytotoxicity profile of drug [20].

Due to its highly lipophilic nature, SN38 can be easily incorporated in the structure of NLCs which can protect the drug from degradation and/or transformation in pH or chemical reactions [21]. In the present study, SN38 loaded NLCs prepared by two different methods were examined for their physiochemical properties, drug release profile, internalization ability, and cytotoxicity against U87MG human glioblastoma cell line [22].

## Materials and methods

### Materials and cells

SN38 was purchased from Knowshine Co. (Shanghai, PR China). Poly vinyl alcohol 88% hydrolyzed, MW 20,000–30,000 Da (PVA) was acquired from Acros Organics (Geel, Belgium). Vitamin E TPGS (γ-tocopherol) was obtained from Jenkem Co. (Beijing, PR China). 3-(4, 5-dimethylthiazol-2-yl)-2,5-diphenyl tetrazolium bromide (MTT) was obtained from Sigma–Aldrich (St. Louis, MO). Dulbecco’s modified Eagle’s medium (DMEM), Fetal bovine serum (FBS) and Trypsin/EDTA were obtained from Life technologies (Grand Island, NY). Oleic Acid (OA) and Cetyl Palmitate (CP) were purchased from Loba Chemie (Mumbai, India). Other chemicals and solvents used in this study were analytical grade. U87MG human glioblastoma cell line was from Pasteur Institute of Iran (Tehran, Iran).

### Preparation **of SN38 loaded NLCs**

SN38 loaded NLCs were prepared using two different methods:

#### Probe ultrasonication hot homogenization method

As the first method, solid/liquid lipids were liquefied and melted using heater stirrer (300 rpm, 40 °C, 15 min). Then SN38 solution in dimethyl sulfoxide (DMSO, 0.5 mg/ml, 100 µL) was added to the lipid mixture. The organic phase was then incorporated into the aqueous phase containing PVA 0.5% using sonic probe homogenizer in an ice bath. After obtaining of milky white colored emulsion, the NLCs were solidified under overnight stirring at room temperature. Prepared NLCs were then collected by centrifugation (100 kDa Amicon^®^, 1800 rpm). The formulation and their corresponding concentrations are shown in Table 1, F_a_ series.

**Table 1:** Formulations for the preparation of NLC by probe ultrasonication hot homogenization (Fa) and modified emulsification solvent evaporation (Fb) techniques

Formulation No.	CP* (wt.%)	Vit E (wt.%)	OA** (wt.%)	*M*^†^ (mg)	*V*^††^ (ml)	Solid: liquid lipid ratio (w/w)
F_a_ series						
F_a1_	55	5	25	200	10	70:30
F_a2_	50	10	25	200	10	70:30
F_a3_	45	5	35	200	10	60:40
F_a4_	40	10	35	200	10	60:40
F_a5_	35	5	45	200	10	50:50
F_a6_	30	10	45	200	10	50:50
F_a7_	16	5	50	200	10	40:60
F_a8_	25	10	50	200	10	40:60
F_a9_	20	5	60	200	10	30:70
F_a10_	15	10	60	200	10	30:70
F_b_ series						
F_b1_	55	5	25	200	10	70:30
F_b2_	50	10	25	200	10	70:30
F_b3_	45	5	35	200	10	60:40
F_b4_	40	10	35	200	10	60:40
F_b5_	35	5	45	200	10	50:50
F_b6_	30	10	45	200	10	50:50
F_b7_	16	5	50	200	10	40:60
F_b8_	25	10	50	200	10	40:60
F_b9_	20	5	60	200	10	30:70
F_b10_	15	10	60	200	10	30:70

*Cetyl palmitate; **Oleic acid; ^†^Total amount of lipid; ^††^Volume of dispersion phase

#### Modified emulsification solvent evaporation technique

In the second method, SN38 loaded NLCs were prepared using a modified emulsification solvent evaporation technique. Briefly, the solid/liquid lipid mixture and SN38 (15% w/w) were separately dissolved in dichloromethane (DCM) and DMSO, respectively. Acquired solutions were then mixed together in constant ratios to form the organic phase. The organic phase was then vigorously mixed with the aqueous phase containing PVA 0.5% using a sonic probe homogenizer (65% amplitude) while the reaction container was located in an ice bath. Homogenization continued for 14 min to achieve a milky white colored emulsion. Organic solvent was then allowed to completely evaporate by stirring at room temperature overnight. Afterward the formed NLCs were collected by centrifugation (100 kDa Amicon^®^, 1800 rpm). The components of each formulation and their corresponding concentrations are reported in Table 1, F_b_ series.

### Nanoparticle characterization

#### Size and morphology

Size, polydispersity index (PDI) and zeta potentials of the prepared NLCs were measured by Malvern Zetasizer ZS (Malvern, UK). The morphology of NPs was investigated by scanning electron microscopy (SEM, KYKY model EM3200, Tokyo, Japan). A suspension of NPs was spread on an aluminum foil and dried at room temperature. The samples were then coated with a thin layer of gold under vacuum in order to be prepared for SEM imaging.

#### Differential scanning calorimetry (DSC)

DSC thermal analysis is widely used to characterize the degree of lipid crystallinity and the polymorphism of the lipids. This technique is based on the difference in melting points and enthalpies of varying lipid types and crystallinities. In the present work, equivalent amounts of SN38 loaded NLC, a physical mixture of NLC/SN38 and, free SN38 were weighed and put in the standard aluminum pans. DSC was then performed utilizing a Mettler DSC 823 unit (Mettler Toledo, Switzerland) equipped with a Julabo thermos cryostat model FT100Y (Julabo labortechnik, Germany) in which indium was used as instrument calibrator. Scanning was carried out in ascending mode (10 °C /min) between 20 and 450 °C under a nitrogen purge, using an empty pan as a reference.

#### Drug loading and entrapment efficiencies

The amount of SN38 loaded in NLCs was determined indirectly using a fluorescence spectroscopy method. Briefly, prepared SN38 loaded NLCs were filtrated by 100 kDa Amicon^®^ Ultra Centrifugal Filter (Merck, Germany) by centrifugation at 3000 g for 50 min. The filtrate was then separated and subjected to fluorometry at λ_exc_ = 407 nm for determination of free amounts of SN38. Entrapment efficiency (EE) and loading efficiency (LE) of SN38 in NLCs were then calculated according to the following equations:

$$EE{\mathrm{\% }} = \frac{{Total\,SN38 - Free\,SN38}}{{Total\,SN38}} \times 100$$

$$LE{\mathrm{\% }} = \frac{{Total\,SN38 - Free\,SN38}}{{NPs\,Mass}} \times 100$$

### In vitro release studies

Standard phosphate buffer saline (PBS) with pH 7.4 was used as the release medium. However, due to the lack of drug dissolution in this environment, evaluation of drug release in this medium was inaccurate. Accordingly, changing the release medium was targeted to overcome the problem. In the first step, Tween 80 (2–4% v/v) was added to the medium, which was not effective. In the next step, PVA 1% (w/v) was incorporated in the medium, which similarly did not provide the desired result. Finally, the addition of albumin 3.5% (w/v) to the phosphate buffer solution allowed the proper dissolution of the drug in release medium and consequently resulted in accurate detection and measurement of the drug.

Following the preparation of the release solution, SN38 loaded NLCs were placed in dialysis bags with 12 kDa cutoff and immersed in this medium. At pre-determined intervals, 5 ml of release medium was removed and replaced with freshly prepared PBS containing 3.5% (w/v) albumin. During the whole test, the sink condition was considered and the concentration of SN38 in the medium was <10% of its saturated concentration in albumin solution (3.5%).

Due to the fluorescence emission overlaps of the SN38 and albumin, it was necessary to separate the attached drug from albumin and extract it by an appropriate solvent to reach an accurate analysis by fluorimeter.

Therefore, hypochlorous acid (HClO 10% v/v) was added to the release samples to precipitate free and SN38 bonded albumins, and centrifuged for 10 min (2500 rpm). Then the sediment was dispersed in DMSO to extract SN38. Subsequently, by centrifuge of this mixture (5000 rpm, 10 mins) the coagulated albumins were separated and the drug concentration in supernatant was evaluated at λexc = 407 nm using a fluorimeter.

### Evaluation of SN38 extraction yield from the release samples

In order to evaluate the extraction method efficacy, the following procedure was conducted. Briefly, two samples were prepared. In the first sample, 100 µL of SN38 solution (0.5 mg/ml in DMSO) was added to the albuminated buffer medium (3.5%) prior to the precipitation of albumin and in the second sample, the same amount of SN38 was added to the supernatant following the albumin precipitation. The albumin precipitation and elimination were conducted as described before. The fluorescence absorption of two samples were evaluated and compared with each other.

### In vitro cell viability assay

The cytotoxicity of free SN38, NLC and NLC-SN38 against U87MG cells was investigated by MTT colorimetric assay. For this measurement, low passage U87MG cells were seeded into 96-well plates about 7000–10000 cells per well. To reach desirable confluence, the cells were incubated for 24 h. Then, cell treatment was carried out at SN38 concentration range of 0.01, 0.1, 1, 10, 50, and 100 μg/ml which was selected according to the release results and previous reports. The cells were incubated for 24, 48, and 72 h at 37 °C and 5% CO_2_. After each time interval, the old medium was replaced with 50 μL of MTT: PBS solution (0.5 mg/ml). The plates were incubated for 2–4 h at 37 °C and 5% CO_2_. Thereafter, DMSO was added to the wells in order to dissolve the water-insoluble formazan crystals. Eventually, the absorbance of the solution was quantified by the microplate reader (BioTEK ELX800, USA) at the wavelengths of 570 and 630 nm as a reference. The experiment was replicated three times. Following formula was used for calculating cell viability:

Viability (%) = Average of absorbance of each well/Average of absorbance of untreated wells × 100.

### In vitro cellular uptake

Cellular uptake of NLCs was investigated by Coumarin-6 (C6) loaded NLCs on U87MG cells. The C6 loaded NLCs preparation method and components were exactly as same as SN38 loaded NLCs, except the C6 concentration which was 50 nM. U87MG cells were seeded into glass-bottom dishes for 24 h. The cells were then treated with C6 loaded NLCs (C6-NLCs) for 2 and 4 h at 37 °C and 5% CO_2_. Cells were then washed three times with PBS to remove unbound C6-NLCs. Afterward, Cells were stained with DAPI (4′,6-diamidino-2-phenylindole), 1 μg/ml, for 5 min in the dark at room temperature and washed three times with PBS to eliminate free DAPI. The cellular uptake of stained C6-NLCs by U87MG cells was investigated using confocal laser scanning microscopy (ECLIPSE Ti, Nikon, Japan).

### Statistical analysis

The MTT assay results were reported as the mean ± SD from four individual experiments. The half maximal inhibitory concentrations (IC_50_) were obtained by SigmaPlot software and compared employing the one-way analysis of variance (ANOVA) test and the outcomes were considered as significantly different when *P* < 0.05.

## Results

### Physiochemical characteristics of prepared NLCs

SN38 loaded NLCs with different solid:liquid lipid ratios employing two methods, probe ultrasonication hot homogenization and modified emulsification solvent evaporation techniques, were prepared. The values of average size, zeta potential, and PDI of each formulation are shown in Table 2. All formulations had a PDI value of <0.5 proposing that all formulations have a narrow size distribution range. The resulted negative zeta potentials, −15 mV to −20 mV, suggest the quite stable formulations. In the probe ultrasonication hot homogenization method, Fa8 and Fa9 formulations, and in modified emulsification solvent evaporation technique, Fb5 and Fb6 formulations demonstrated the lowest particle size; 2 and 24 h, respectively after preparation. Therefore, these formulations were chosen for further analysis.

**Table 2:** The obtained size and PDI of prepared NLCs after 2 and 24 h. Fa refer to probe ultrasonication hot homogenization method, Fb refers to modified emulsification solvent evaporation method

Formulation No.	2 h		24 h	
	Size (nm)	PDI	Size (nm)	PDI
F_a_ series				
F_a1_	220.40 ± 5.21*	0.26 ± 0.01	245.43 ± 5.10	0.31 ± 0.03
F_a2_	211.61 ± 7.22	0.29 ± 0.10	234.40 ± 6.32	0.28 ± 0.03
F_a3_	199.32 ± 5.40	0.26 ± 0.08	270.71 ± 3.91	0.29 ± 0.01
F_a4_	200.62 ± 4.71	0.25 ± 0.07	227.61 ± 5.11	0.24 ± 0.02
F_a5_	211.80 ± 3.93	0.24 ± 0.02	244.82 ± 3.90	0.26 ± 0.01
F_a6_	190.54 ± 2.72	0.23 ± 0.01	201.12 ± 4.23	0.32 ± 0.03
F_a7_	164.81 ± 3.70	0.20 ± 0.03	172.13 ± 5.13	0.38 ± 0.02
F_a8_	156.23 ± 5.10	0.26 ± 0.01	167.40 ± 3.21	0.24 ± 0.02
F_a9_	159.50 ± 2.70	0.26 ± 0.02	181.80 ± 4.70	0.24 ± 0.02
F_a10_	181.11 ± 2.42	0.21 ± 0.01	186.12 ± 3.64	0.21 ± 0.01
F_b_ series				
F_b1_	215.42 ± 5.10	0.26 ± 0.03	234.40 ± 5.30	0.28 ± 0.02
F_b2_	209.41 ± 6.31	0.27 ± 0.02	230.71 ± 3.80	0.27 ± 0.03
F_b3_	195.71 ± 3.92	0.25 ± 0.03	228.60 ± 5.23	0.26 ± 0.02
F_b4_	190.63 ± 5.12	0.24 ± 0.04	224.83 ± 3.92	0.26 ± 0.02
F_b5_	148.10 ± 2.71	0.14 ± 0.02	160.01 ± 2.71	0.15 ± 0.02
F_b6_	144.02 ± 3.22	0.22 ± 0.03	155.41 ± 4.22	0.24 ± 0.03
F_b7_	172.03 ± 2.72	0.40 ± 0.01	244.81 ± 3.93	0.36 ± 0.00
F_b8_	166.02 ± 6.32	0.33 ± 0.02	201.11 ± 4.23	0.34 ± 0.03
F_b9_	211.80 ± 3.91	0.24 ± 0.02	172.10 ± 5.11	0.37 ± 0.01
F_b10_	252.10 ± 4.20	0.22 ± 0.02	181.12 ± 2.40	0.24 ± 0.03

*Mean ± SD (*n* = 3)

### Drug loading and entrapment efficiencies

Selected formulations with the lowest particle size and PDI were investigated for LE and EE%. As presented in Table 3, F_b5_ (lipid phase: cetyl palmitate 7 mg, Vit. E TPGS 1 mg, oleic acid 9 mg. aqueous phase: PVA 0.5% w/v) prepared by modified emulsification solvent evaporation technique had the highest loading and entrapment efficiencies and therefore, it was selected for further experiments.

**Table 3:** Comparing LE and EE% of selected formulations Fa represents the probe ultrasonication hot homogenization method, Fb represents the modified emulsification solvent evaporation method

Formulation No.	LE%	EE%	Zeta potential
F_a8_	5.51 ± 0.44*	46.06 ± 1.02	−18.02 ± 0.45
F_a9_	6.01 ± 0.71	51.37 ± 0.96	−18.33 ± 0.51
F_b5_	9.50 ± 0.32	81.36 ± 0.69	−16.04 ± 0.60
F_b6_	9.42 ± 0.82	76.38 ± 1.12	−17.10 ± 0.77

*Mean ± SD (*n* = 3)

### Characterization of SN38 loaded NLCs

**Fig. 1:**
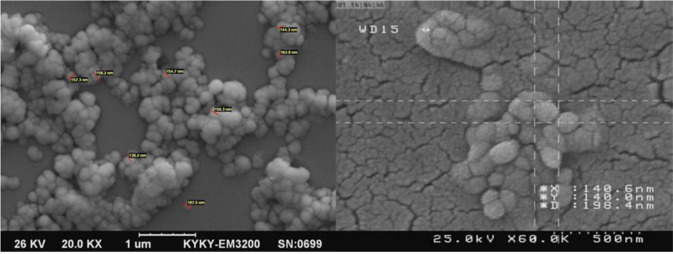
SEM images of NLC nanoparticles loaded with SN38

Figure 1 demonstrates the SEM results of Fb5 formulation. As can be observed, the morphologies of NLCs are all semi spherical in shape and similar in size and also the sizes measured by SEM were comparable to those obtained by DLS method. The observed aggregation may be due to the preparation process for SEM imaging.

**Fig. 2:**
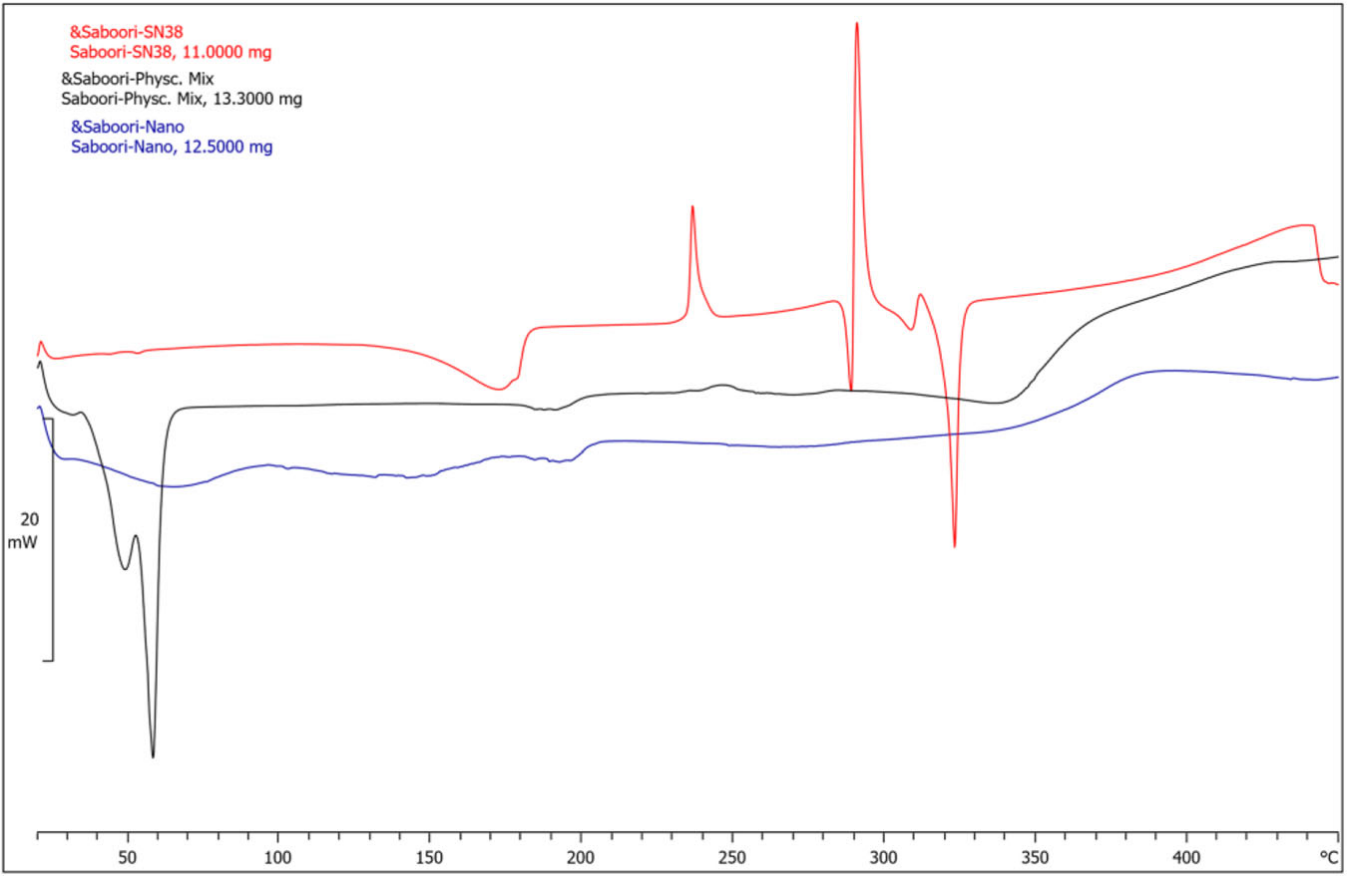
DSC thermograms of free SN38 (red), physical mixture of SN38 and lipid (black), and SN38 loaded NLC nanoparticles (blue)

The DSC thermograms of SN38, NLCs containing SN38, and physical mixture of SN38 and lipid are shown in Fig. 2. The endothermic glassy transition temperature for drug loaded NLCs appeared around 40–60 °C. In addition, SN38 demonstrated four sharp melting endothermic peaks at 282 °C and 318 °C and exothermic peaks at 235 °C and 286 °C corresponding to its melting point in the crystalline state (red thermogram). The peak around 40–60 °C in the thermogram of the physical mixture of lipids and drug (black thermogram) is related to the melting point of TPGS (37–41 °C) and cetyl palmitate (54 °C). The drug peak in the mixture has shifted to 340–270 °C and significantly diminished which can be explained by solvation of drug in lipids or interactions due to heating. The absence of SN38 peaks in thermogram of drug loaded NLCs (blue thermogram) suggests an amorphous and non-crystalline structure of SN38 in these particles and proves appropriate distribution of the drug in NLCs.

### In vitro drug release

In order to analyze the released drug, SN38 was extracted from the release medium, which was containing albumin. Based on the pre-study results, the yield of the extraction method was about 70% and the related correction factor was applied to the all released SN38 concentrations, accordingly.

**Fig. 3:**
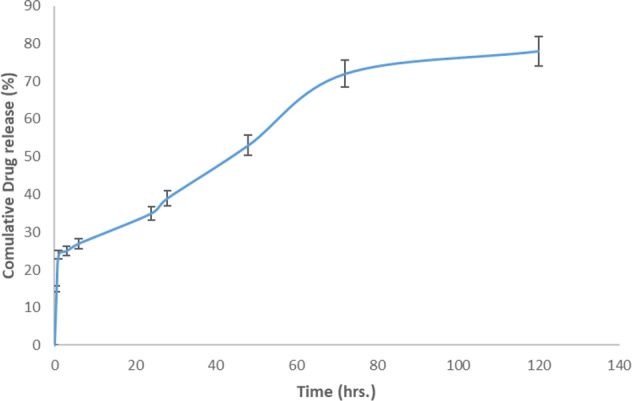
The in vitro release profile of SN38 released from the NLC in PBS containing 3.5% albumin, pH = 7.4, (*n* = 3)

SN38 cumulative released profile is displayed in Fig. 3. The drug release pattern followed a tri-phasic diagram consisting of a burst release within the first hour, a slower release up to 72 h and a near steady state phase up to the end of 120 h. As shown in Fig. 3, about 20% of SN38 was released during the first phase which may attributed to the portion of SN38 molecules bounded on the surface of NLCs. The amount of released drug during the burst release is of utmost importance as it is tightly correlated with formulations toxicity and also efficacy. Following the burst release the slower second phase begins which may relate to the SN38 molecules entrapped close to the surface. About 50% of the loaded drug was released within this phase. During the third following phase about 10% of the remained drug was released, which is corresponded to the fully entrapped drugs. Thus, the two final phases proposed a slow releasing rate for SN38 during the test span.

### In vitro cytotoxicity

**Fig. 4:**
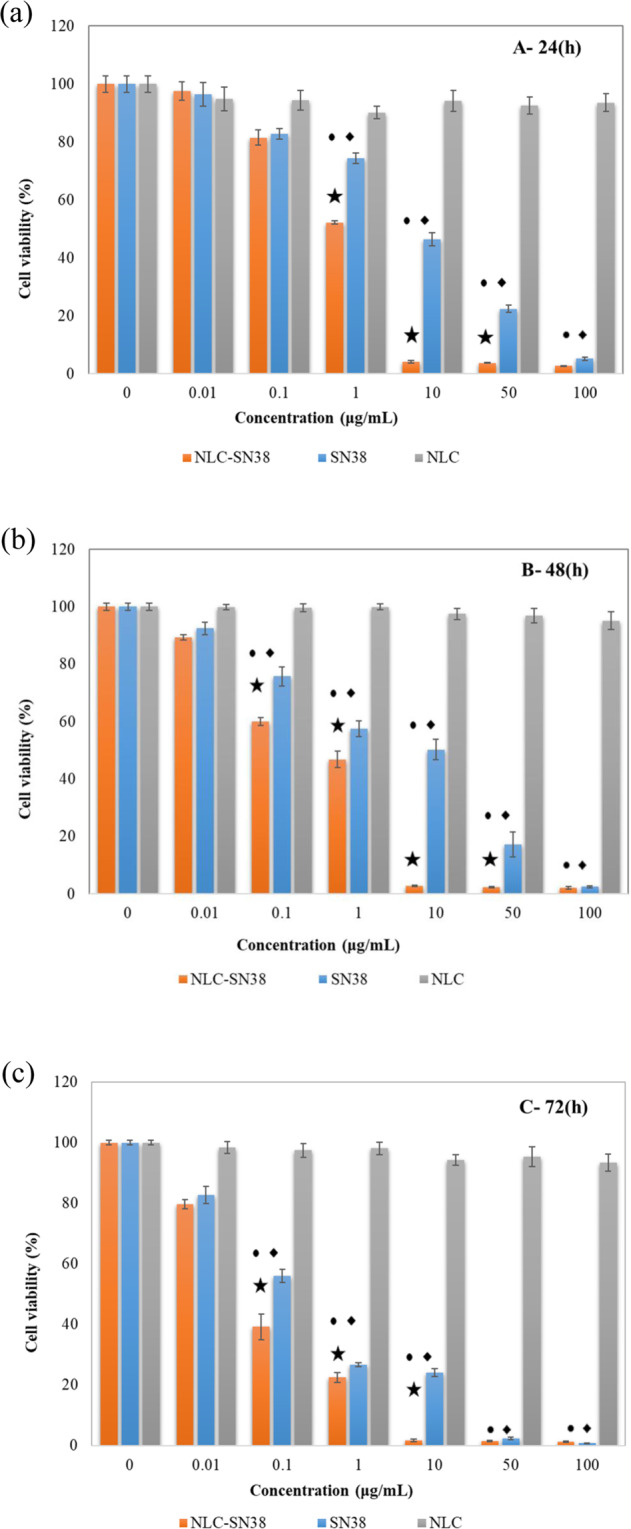
(**a**) MTT assays of NLC-SN38 (orange), SN38 (blue), and NLC (gray) in 24 h, (**b**) 48 h and (**c**) 72 h after treatment at the concentration range of 0 as control to 100 μg/mL against U87MG cells. “•” indicates the significancy of control vs. SN38 (*P* < 0.001), “♦” indicates the significancy of control vs. NLC-SN38 (*P* < 0.001), and “★” indicates the significancy of SN38 vs. NLC-SN38 (*P* < 0.001) (*n* = 3)

The cytotoxicity profile of free NLC as carriers, free SN38 and SN38 loaded NLC in the range of 0.01–100 mM were studied by MTT assay on U87MG glioblastoma cell lines. The cell viability after 24-, 48-, and 72-hour treatment with mentioned formulations are displayed in Fig. 4 a–c, respectively.

As depicted in Fig. 4a, after 24-hours incubation, with increasing SN38 and SN38 loaded NLC concentration, the cell viability was decreased. At the concentrations higher than 1 µg/ml, drug loaded NLCs demonstrated a significant higher cytotoxicity compared to the free drug. IC_50_ values for SN38 loaded NLCs after 24 h incubation was about 2.12 µg/ml which was significantly lower than that of free SN38, 8.44 µg/ml (*P* < 0.05). This result revealed that the prepared SN38 loaded NLCs, was more effective than the free drug after first 24 h. No significant cytotoxicity was observed for the drug-free (blank) nanolipids even in high concentrations over the 24-hours.

After 48-hours, the measured IC_50_ values for the SN38 loaded NPs and the free drugs were 0.32 μg/ml and 10.15 μg/ml, respectively (Table 4). As shown in Fig. 4b, after 48 h, SN38 loaded NLCs still have higher cytotoxicity effect rather than free SN3 (*P* < 0.05).

Finally, by increasing incubation time up to 72-hours, a falling trend was similarly observed in the number of live cells (Fig. 4c). The measured IC_50_ values for the NP containing SN38 was 0.06 μg/ml. However, for the free drug, it was 0.38 μg/ml, which indicated the effectiveness of NLC-SN38 in comparison with the free SN38. As can be seen the blank NLCs indicated no detectable toxicity over 48 and 72-hour incubations, which means they can be applied as biocompatible nanocarriers either in drug delivery systems or in other bio applications. The IC_50_ values of free SN38 and NLC-SN38 after 24, 48, and 72 h were given in Table 4.

**Fig. 5:**
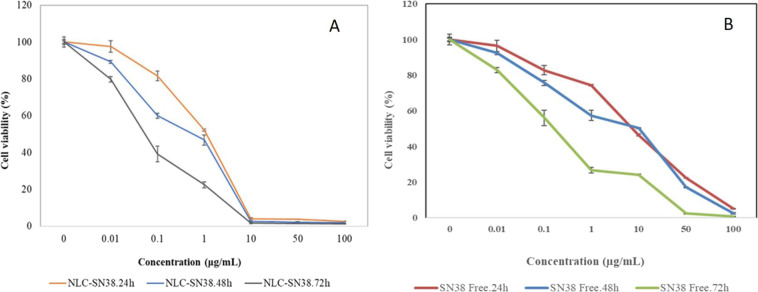
**A** The profile of cytotoxicity vs. concentration of NLC-SN38 at 24 (orang line), 48 (blue line) and 72 h (gray line) after cell, U87MG, treatment. **B** The profile of cytotoxicity vs. concentration of free SN38 at 24 (red line), 48 (blue line) and 72 h (green line) after cell, U87MG, treatment (*n* = 3)

According to the Fig. 5, NLC-SN38 cytotoxicity trend indicated a time dependent manner following 24-, 48-, and 72-hour incubations. By increasing incubation time, the cytotoxicity effect was significantly increased (*P* < 0.05) which confirm the in vitro sustain release profile (Fig. 3). Moreover, the cell viability has been dramatically decreased in concentrations above 0.10 μg/ml which also affirm the effectiveness of the prepared SN38-NLC.

### In vitro cellular uptake

**Fig. 6:**
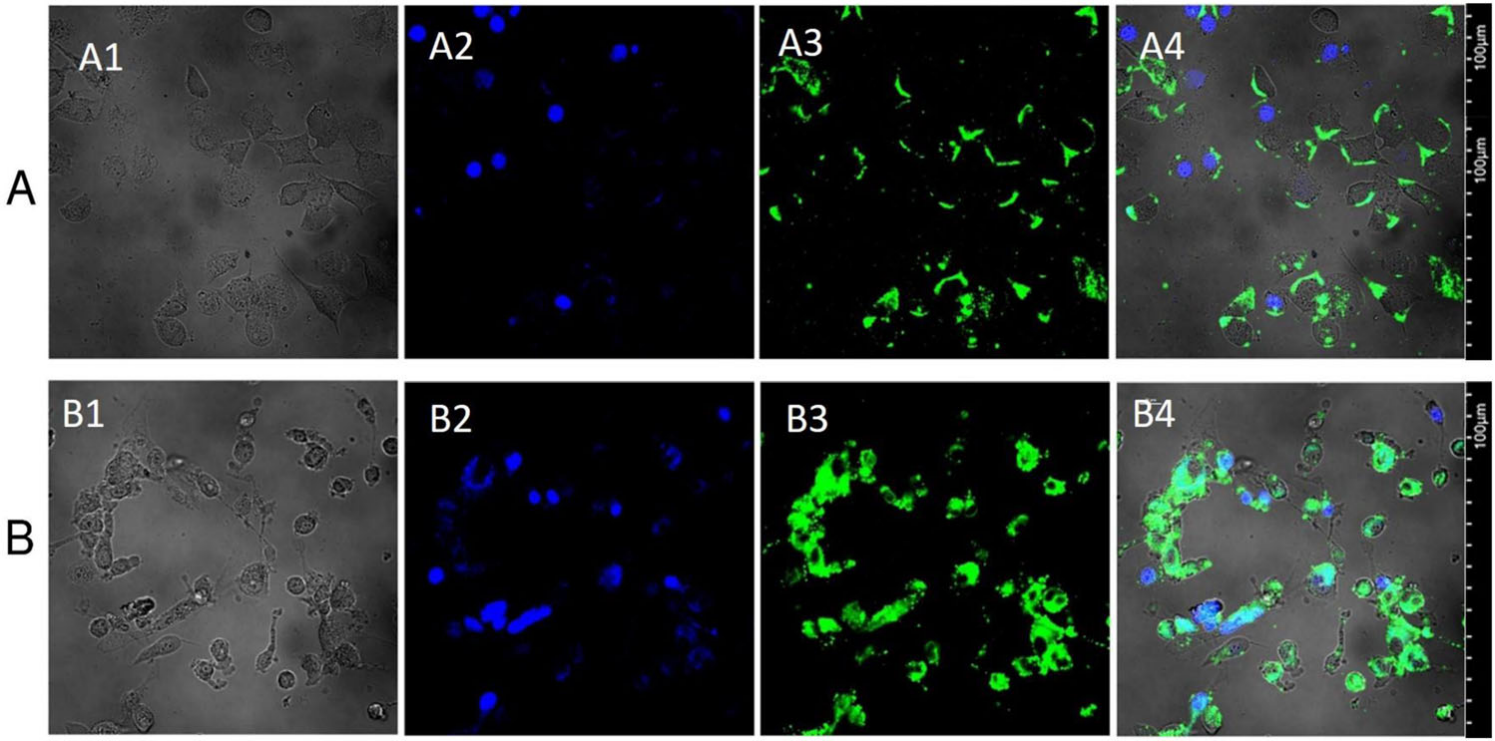
In vitro cellular uptake of C6-NLCs in U87MG glioma cell lines, after (**A**) 2-h and (**B**) 4-h incubations. A1 and B1: optical microscope images, A2 and B2: cellular nucleus colored with Dapi dye (blue), A3 and B3: the up taken C6-NLCs into the cell cytoplasm (green), A4 and B4: merged images.

Confocal microscopy was employed for assessing the in vitro cellular uptake of NLCs by U87MG glioblastoma cells. C6 dye was applied as a fluorescent marker for visualizing the NLC cellular uptake. To determine the effect of incubation time on the cellular uptake of C6 loaded NLCs, cells were incubated with 1 μg/ml of NLCs for 2 and 4 h. During the first 2 h (Fig. 6A), low cytoplasmic uptake was detected while a remarkable NP adsorption on the cell surfaces was observed. However, increasing the incubation time to 4 h resulted in a notable NLC uptakes by U87MG cells (Fig. 6B).

## Discussion

During the current decades the effect of numerous anticancer formulations has been investigated based on their capability of reducing tumor growth and inducing the least cytotoxicity to normal adjacent cells [23]. Nevertheless, most of the current approved chemotherapeutic agents suffer from the lack of selective toxicity [24]. Tumor tissues possess leaky vasculature with pores of about 200–600 nm in size and insufficient lymphatic drainage. This phenomenon allows NPs to easily enter to the tumor microenvironment and stay there for a long period which in turn, results in enhanced drug transportation to the site of action and reduction in adverse effects [25].

Vitamin E (TPGS) is a multipurpose component employed in NLC formulations. As the cancer cells possess a higher metabolic rate compare to normal ones, the density of TPGS receptors on the surface of cancer cells are much higher. Thus, TPGS can play a targeting moiety role. Furthermore, it has been proved that TPGS is capable to increase the solubility of drugs, enhance the drug cytotoxicity and inhibit P-glycoproteins pumping on the surface of tumor cells [26]. Inducing apoptosis in cancer cells has been also demonstrated by TPGS [27]. Accordingly, the use of TPGS in this study may enhance the apoptosis via targeted delivery; however, further works is needed to prove this point.

As an active metabolite of irinotecan, SN38 have shown to possess approximately 100–1000 times more strength in inhibiting the activity of topoisomerase I [28, 29]. Due to the high potency, multiple studies have addressed the beneficial effects of SN38 in treatment of various cancers some of which include gastric, ovarian and cervical cancers, colorectal carcinoma and also brain tumors [30]. But, SN38 has low solubility in pharmaceutical solvents which seriously limits its application in therapeutic purposes. Moreover, the additional challenges is the low metabolism rate of the pro-drug to the therapeutic active form, SN38 (1–9%) [11, 31]. Therefore, here we prepared an NLC formulation of SN38 to achieve better solubility and overcome to other limitations associated with direct application of SN38.

Among different drug delivery formulations, lipid vesicles including SLNs and NLCs appears to be one of the most promising ones for controlled drug delivery. Since their surface resemble those of cell membrane lipids, they do not cause any harm during administration to the body and are simply incorporated into the cells [16, 32]. Specific advantages of applying lipid base vesicles for delivery of camptothecin and its analogues including irinotecan and SN38 in to cancer cells include: enhancing internalization of drug molecules, reducing several systemic toxicities and increasing solubility of drug molecules in biological fluids. Nevertheless, application of these systems still suffers from some limitations such as non-selectivity to the target cancer cells which can be overcome by surface modification with specific targeting moieties [33, 34].

Overall, drugs reside between fatty acid chains, lipid layers or in the amorphous clusters located in crystalline deficits of NLC matrix. In SLNs, lipids become crystallized in α and β‘ forms during high energy lipid alterations immediately after their preparation. But with the time, lipid molecules underwent a reforming process, through which low energy β and βi crystals are formed. Formation of this full crystalline structure results in leakage of drugs from SLNs. These result in low loading capacity and high probability of drug leakage and instability of SLNs. Therefore, NLCs were developed to overcome these disadvantages. In preparation of these particles, a mixture of both solid and liquid lipids (oils) is utilized to make lipid matrix deficit as much as possible. This results in maximizing drug loading and inhibition of drug leakage during the storage period which can perfectly explain the high EE% obtained in our results [35].

Probe ultrasonication hot homogenization and modified emulsification solvent evaporation techniques are two main approaches for preparation of NLCs [36]. Previous studies have reported that one of the major drawbacks in the probe ultrasonication hot homogenization method is the reduced drug loading and probable lipids and drugs degradation due to increasing the temperature [33]. However, some sources have pointed out that this method will partly reduce the size of NPs [37]. Our experiments on the NLC-SN38 formulation, showed that the samples made by solvent evaporation-emulsification with the appropriate formulation had comparable sizes with statistically significant higher LE and EE% (*P* < 0.05) compared with the hot homogenization method. Although, various reports have shown high burst release of drugs from the small sized NLCs, our prepared NPs presented <20% burst release, which demonstrates the low adsorbed SN38 on the particle surface and effective drug loading into the NLC structure [38].

According to the previous reports, solvent evaporation method commonly results in a faster drug releasing profile from NLCs in comparison with melt-emulsification technique. In melting evaporation technique, the melting temperature for drug and liquid are different (45–60 °C for Vit E and Oleic acid and 240–250 °C for SN38), consequently, the drugs with higher melting points will be crystallized first during the cooling process and form a drug enriched center. This usually causes lower drug release rates which can be appropriate in controlled release systems [39, 40]. However, in cancer therapy immediate release following the target site achievement is more favorable. On the other word, a reduced burst following by a slow release will efficiently deliver the cytotoxic to the tumor with minimal side effects from high drug concentrations. The obtained release results in this study (Fig. 3) indicated a proper profile with less than 20% burst release.

Based on outcomes, all formulations showed negative surface zeta potential values. Zeta potential represents the degree of repulsion forces between adjacent particles in a dispersion medium. As these forces increase, the electrostatic repulsions between close particles elevate and result more stable NLC dispersion. The values of above +20 mV or less than −20 mV commonly guarantees an appropriate physical stability for dispersions [38]. Regarding Table 4, F_b5_ formulation possessed a zeta potential of −16 mV which may result in an acceptable stability of the NPs and prevent from aggregation [41].

**Table 4:** The IC_50_ values of free SN38 and NLC-SN38 determined on U87MG glioblastoma cancer cell line

	Incubation time		
	24 h	48 h	72 h
Free SN38	8.44 ± 0.90*	10.15 ± 1.23	0.38 ± 0.51
NLC-SN38	2.12 ± 0.57	0.38 ± 0.05	0.06 ± 0.02

*Mean ± SD (*n* = 3)

SEM images represented NPs with appropriate polydispersity index, uniform and spherical shape (Fig. 1). The size reported with this method is according to the size reported by Zetasizer. The results of this study correspond to previous studies on NLC structures [42].

To access the best simulated drug release medium as same as the body fluid, many studies have been done previously [43]. Based on the lipid nature of NLCs and SN38, according to the previous researches, pH 7.4 was selected [44]. In some studies, the amount of drug released was quantified by UV-Visible spectroscopy, but unfortunately, this technique was not practically appropriate in our study due to the lipid interference peaks with the drug. Thus, the fluorescence spectroscopy method was replaced. In order to improve the release of SN38 from the nano-structure albumin was chosen as an assistant agent. Noteworthy, it is a novel approach in in vitro release analysis of SN38 loaded formulation. Our results revealed a continuous and ascending release of the drug following an initial burst which was compatible with similar studies in this field [45].

Regarding to the cytotoxicity profile of free SN38 and SN38 loaded NLCs, no significant toxicity was observed at low doses (0.1 and 0.01 μg/ml) after 24 h exposure. As SN38 is a drug with mechanism of action of inhibiting topoisomerase I, this drug affects mostly during the S phase of cell cycle [46]. The doubling time for U87MG has been estimated to be about 1.3 days (32 h) [47]. So, as the cells had not reached the S phase after the first 24 h treatment, it is not surprising that the changes were not significant. Contrarily, after 48 h and 72 h, free SN38 and SN38 loaded NLCs demonstrated a significantly increased cytotoxicity compared to 24 h interval. The enhanced cytotoxicity of SN38 loaded NLCs compared to free SN38 may be attributed to the enhanced cellular uptake which was observed by confocal microscopy. However, more cellular experiments are required to confirm this point [48, 49].

The possible mechanism underlying the enhancement of uptake and subsequent cytotoxicity of camptothecin applying lipid vesicles may be attributed to the following reasons: first, these carriers may act as a reservoir for camptothecin, releasing it over time for further uptake by cells although a few studies have now repealed this theory. Second, they may be taken in to the cell through the endocytosis pathways [50].

## Conclusion

In current study, we developed an appropriate NLCs containing SN38 as a potential anticancer formulation to overcome the issues such as drugs solubility, instability, and delivery to the tumor cells. The prepared NLCs exhibited a triphasic drug release profile which reached about 75% at the end of the test span. Accordingly, the designed release medium was efficient for the diffusion of the hydrophobic drug (SN38) from the hydrophobic carrier (NLC) and the extraction method was a proper sample preparation for an accurate analysis. To conclude, the proper cellular uptake and significant more cytotoxicity compared to the free drug indicates the potentials of the prepared NLCs to be introduced as a novel SN38 delivery system for treatment of glioblastoma.
